# Treatment‐Related Adverse Events and Associated Outcomes in Patients With Advanced Urothelial Carcinoma Treated With Enfortumab Vedotin: Analysis of the UNITE Study

**DOI:** 10.1002/cam4.71284

**Published:** 2025-10-01

**Authors:** Amanda Nizam, Charles B. Nguyen, Jinju Li, Emily C. Zabor, Pavlos Msaouel, Cindy Y. Jiang, Omar Alhalabi, Eugene Oh, Matthew P. Davidsohn, Ilana B. Epstein, Dimitra Rafailia Bakaloudi, Rafee Talukder, Tanya Jindal, Amy K. Taylor, Michael J. Glover, Ali Raza Khaki, Emily Lemke, Hannah Mabey, Bashar Abuqayas, Albert Jang, Jason R. Brown, Sean T. Evans, Cameron Pywell, Arnab Basu, Mehmet A. Bilen, Pedro C. Barata, Yousef Zakharia, Matthew I. Milowsky, Deepak Kilari, Christopher J. Hoimes, Sumit A. Shah, Hamid Emamekhoo, Nancy B. Davis, Shilpa Gupta, Petros Grivas, Joaquim Bellmunt, Matthew T. Campbell, Ajjai S. Alva, Vadim S. Koshkin

**Affiliations:** ^1^ Department of Hematology and Medical Oncology Cleveland Clinic Taussig Cancer Institute Cleveland Ohio USA; ^2^ Department of Hematology and Oncology University of Michigan Rogel Comprehensive Cancer Center Ann Arbor Michigan USA; ^3^ Department of Biostatistics University of Michigan Medical School Ann Arbor Michigan USA; ^4^ Department of Quantitative Health Sciences Cleveland Clinic Lerner Research Institute Cleveland Ohio USA; ^5^ Department of Genitourinary Medical Oncology The University of Texas MD Anderson Cancer Center Houston Texas USA; ^6^ Division of Cancer Medicine The University of Texas MD Anderson Cancer Center Houston Texas USA; ^7^ Department of Medicine University of Michigan Medical School Ann Arbor Michigan USA; ^8^ Genitourinary Oncology Program Dana‐Farber Cancer Institute‐IMIM Research Lab Boston Massachusetts USA; ^9^ Division of Hematology and Oncology, Department of Medicine University of Washington Seattle Washington USA; ^10^ Division of Hematology/Oncology Baylor College of Medicine Houston Texas USA; ^11^ Department of Hematology/Oncology, University of California San Francisco Helen Diller Family Comprehensive Cancer Center San Francisco California USA; ^12^ Department of Medicine University of Wisconsin Carbone Cancer Center Madison Wisconsin USA; ^13^ Division of Oncology Stanford University Stanford California USA; ^14^ Division of Hematology and Oncology Froedtert and the Medical College of Wisconsin Milwaukee Wisconsin USA; ^15^ Department of Medicine, Division of Oncology University of North Carolina School of Medicine Chapel Hill North Carolina USA; ^16^ Department of Medicine University of Iowa Holden Comprehensive Cancer Center Iowa City Iowa USA; ^17^ Department of Medicine Tulane University School of Medicine New Orleans Louisiana USA; ^18^ Department of Medicine Case Western Reserve University/University Hospitals Seidman Cancer Center Cleveland Ohio USA; ^19^ Department of Hematology and Medical Oncology Winship Cancer Institute of Emory University Atlanta Georgia USA; ^20^ Department of Medicine University of Alabama at Birmingham O'neal Comprehensive Cancer Center Birmingham Alabama USA; ^21^ Department of Medicine Duke Cancer Institute Durham North Carolina USA; ^22^ Department of Medicine, Division of Hematology and Oncology Vanderbilt University Medical Center Nashville Tennessee USA; ^23^ Clinical Research Division Fred Hutchinson Cancer Center Seattle Washington USA

**Keywords:** adverse events, antibody–drug conjugates, enfortumab vedotin, treatment‐related toxicity, urothelial carcinoma

## Abstract

**Introduction:**

Enfortumab vedotin‐ejfv (EV), an antibody–drug conjugate approved for advanced urothelial carcinoma (aUC), has limited real‐world safety data and no validated predictive biomarkers. Retrospective studies suggest higher objective response rates (ORR) among patients developing EV‐related dermatologic toxicities, but survival implications remain unclear. This analysis assessed the safety of EV monotherapy and the impact of dermatologic and neurologic toxicities on efficacy outcomes in a multi‐institutional cohort.

**Materials and Methods:**

UNITE is a multicenter, retrospective study across 16 US sites of patients with aUC treated with targeted agents, including EV. Primary endpoints were the incidence of EV treatment‐related adverse events (TRAEs, any grade) and TRAE‐related treatment modifications. Secondary endpoints included a comparison of ORR, progression‐free survival (PFS), and overall survival (OS) between patients with and without dermatologic toxicities and neuropathy. To reduce immortal time bias, Cox regression models incorporated TRAEs as time‐dependent covariates to adjust for EV treatment duration as well as Eastern Cooperative Oncology Group performance status (ECOG PS), hemoglobin, and liver metastases.

**Results:**

Between 2018 and 2023, 485 patients with aUC received > 1 dose of EV monotherapy. Most (67%) received > 2 prior therapy lines. Any grade TRAEs occurred in 77%, most frequently neuropathy (36%) and dermatologic toxicities (27%); 57% required treatment modification and 21% discontinued EV, primarily due to neuropathy (9%) and fatigue (3%). Patients with dermatologic toxicities and neuropathy had higher ORR (56% vs. 42%; *p* = 0.007 and 64% vs. 35%; *p* < 0.0001, respectively). Neuropathy was independently associated with improved OS (HR 0.66; 95% CI 0.50–0.87; *p* = 0.002); dermatologic TRAEs were not associated with PFS or OS benefit.

**Conclusions:**

EV safety outside clinical trials was consistent with prior reports, with no new signals. Treatment modifications were most often due to neuropathy, dermatologic toxicities, and fatigue. Both dermatologic toxicities and neuropathy correlated with higher ORR, but only neuropathy was independently associated with improved OS.

## Abstract 2

Enfortumab vedotin‐ejfv (EV) is an approved therapy for advanced urothelial carcinoma (aUC), but real‐world safety data remain limited. In the UNITE multicenter retrospective cohort study, EV monotherapy demonstrated a toxicity profile consistent with previously reported safety data from pivotal EV trials, without new safety concerns, though with slightly higher rates of treatment discontinuation due to adverse events. Both dermatologic toxicities and neuropathy were associated with improved response rates, but only neuropathy was independently associated with longer overall survival after adjustment for EV treatment duration and established poor prognostic factors in aUC.

**Abbreviations**
ADC: antibody–drug conjugate
ALC: absolute lymphocyte count
ANC: absolute neutrophil count
aUC: advanced urothelial carcinoma
CI: confidence interval
CR: complete response
CTCAE: Common Terminology Criteria for Categorization of Adverse Events
ECOG PS: Eastern Cooperative Oncology Group performance status
eGFR: estimated glomerular filtration rate
EMR: electronic medical record
EV: enfortumab vedotin‐ejfv
FGFR: fibroblast growth factor receptor
Hgb: hemoglobin
HR: hazard ratio
ICI: immune checkpoint inhibitor
ITT: intention‐to‐treat
KPS: Karnofsky performance status
MMAE: monomethyl auristatin E
NE: not evaluable
NLR: neutrophil‐to‐lymphocyte ratio
ORR: observed response rate
OS: overall survival
PD: progression of disease
PFS: progression‐free survival
PR: partial response
SD: stable disease
SJS: Stevens‐Johnson Syndrome
TEN: toxic epidermal necrolysis
TRAE: treatment‐related adverse event
UNITE: Urothelial Cancer Network to Investigate Therapeutic Experiences
WBC: white blood cell count

## 1 Introduction

Advanced urothelial carcinoma (aUC) is associated with a poor prognosis and a five‐year survival rate of less than 10% [1]. The treatment armamentarium for aUC has grown rapidly over the past 5 years with the approval of the fibroblast‐targeted growth receptor (*FGFR*) *1–4* tyrosine kinase inhibitor erdafitinib, novel antibody–drug conjugates (ADCs) including enfortumab vedotin‐ejfv (EV) and sacituzumab govitecan‐hziy (FDA approval voluntarily withdrawn in November 2024), switch maintenance therapy with avelumab, and most recently, the combinations of EV with pembrolizumab and gemcitabine, cisplatin, and nivolumab for the first‐line treatment of aUC [2, 3, 4, 5, 6, 7, 8, 9, 10, 11]. EV is an ADC comprised of a fully humanized IgG antibody directed against the transmembrane cell adhesion molecule Nectin‐4 that is conjugated via a protease‐cleavable linker to monomethyl auristatin E (MMAE), a microtubule‐inhibiting chemotherapy payload [12, 13].

EV received accelerated approval by the United States Food and Drug Administration (FDA) for the treatment of aUC in December 2019 based on results from the phase II single‐arm EV‐201 trial, followed by full approval in 2021 based on the positive confirmatory randomized phase 3 EV‐301 clinical trial [4, 5, 14, 15]. The EV‐301 trial investigated EV versus investigator's choice of single‐agent chemotherapy (docetaxel, paclitaxel, or vinflunine in Europe) in 608 patients with aUC previously treated with platinum‐based chemotherapy and immune checkpoint inhibitor (ICI) therapy [5]. EV demonstrated longer overall survival (OS) of 12.9 months (95% CI 10.6–15.2) vs. 9.0 months (95% CI 8.1–10.7) with chemotherapy (HR 0.70; 95% CI 0.56–0.89; *p* = 0.001) [5, 16].

While EV has demonstrated impressive efficacy in aUC, it is associated with notable toxicities, including an FDA black box warning for severe and potentially fatal skin reactions such as Stevens‐Johnson Syndrome (SJS) and toxic epidermal necrolysis (TEN) [12, 16]. In the EV‐301 trial, 64% of patients developed a TRAE that resulted in EV drug interruption; 34% experienced a TRAE necessitating dose reduction; 15% of patients discontinued EV due to TRAEs. The most common TRAEs leading to any treatment modification (dose reduction, interruption, or discontinuation) were peripheral neuropathy, dermatologic toxicities, and fatigue [5, 16]. The incidence of EV monotherapy TRAEs and associated treatment modifications has not been fully characterized outside of clinical trial settings.

Despite ongoing investigation, biomarkers associated with response to EV have not been validated [17, 18, 19]. Post hoc exploratory analyses from pooled data from pivotal EV monotherapy trials, including EV‐301, demonstrated that higher EV dose intensity during the initial two treatment cycles was associated with increased objective response rates (ORR) and that EV exposure correlated directly with the incidence of TRAEs [20]. Vlachou et al. observed improved ORR among patients who developed dermatologic toxicities while on EV in a single‐institution retrospective cohort study [21]. Furthermore, a subset analysis of the phase III MPACT trial of nab‐paclitaxel and gemcitabine in metastatic pancreatic adenocarcinoma reported a longer median OS in patients who developed higher‐grade vs. lower‐grade peripheral neuropathy [22]. To our knowledge, the relationship between specific TRAEs and survival outcomes in patients treated with EV monotherapy has not been reported.

Urothelial Cancer Network to Investigate Therapeutic Experiences (UNITE) is a multicenter retrospective cohort study including patients with aUC treated with novel systemic agents such as EV [23]. UNITE previously reported robust efficacy outcomes in a large cohort of patients with aUC treated with EV that were consistent with clinical trial data and also extended to patient subsets that would have been excluded from clinical trials [23]. The analysis presented herein reports the toxicity profile of EV, TRAE‐associated treatment modifications, and efficacy outcomes in patients with specific toxicities of interest, mostly outside of a clinical trial setting.

We hypothesized that the toxicity profile of EV and corresponding treatment modifications observed in this study would align with those reported in clinical trials, and that the occurrence of dermatologic toxicities and neuropathy would be associated with higher ORR, potentially translating into improved survival outcomes.

## 2 Materials and Methods

### 2.1 Study Design and Patients

The UNITE multicenter, retrospective cohort study includes patients with locally advanced or metastatic bladder or urinary tract cancer with any histology. All included patients must have received at least one dose of EV alone or in combination with other drugs, whether as standard of care or as part of a clinical trial whose results have already been reported. Eligible patients must have also had available clinical, pathologic, and radiographic data.

The data cutoff date for this analysis was April 1, 2023. This analysis included patients treated with EV monotherapy who were assessed for EV‐related TRAEs, defined as “definitely,” “probably,” or “possibly” attributed to EV as assessed by the treating physician. Patients who received EV in combination with other agents were excluded from this analysis. A CONSORT flow diagram of the overall study is illustrated in Figure S1. Efficacy outcomes were compared between patients with and without TRAEs of special interest, including dermatologic toxicities and neuropathy. CONSORT flow diagrams of these analyses are illustrated in Figures S2 and S3, respectively.

### 2.2 Data Collection

This study met the Declaration of Helsinki principles and was approved by each participating institution's Institutional Review Board as minimal risk research for which a waiver for informed patient consent was granted. Demographic, clinical, treatment, and investigator‐assessed toxicity‐related data were abstracted via retrospective review of electronic medical records (EMR) and collected following each institution's institutional review board‐approved protocol. All data were reported in a deidentified manner and entered in a secure central REDCap database housed at the University of Michigan in accordance with data use agreements approved by each institution's institutional review board and legal departments. All protected health information was excluded.

### 2.3 Outcomes

The primary analyses were calculations of the incidence of TRAEs and TRAE‐related treatment modifications that occurred after at least one dose of EV. TRAE classification, attribution, and grading were based on the Common Terminology Criteria for Adverse Events version 5.0 (CTCAE version 5.0) and determined by the treating physician, if available [24]. If not specified by the treating physician in the EMR, TRAEs were determined retrospectively by local investigators at participating sites using CTCAE version 5.0 criteria. Treatment modifications were classified as the occurrence of drug interruption, dose reduction, or drug discontinuation and were determined by review of clinical notes and EV administration history in the EMR.

Given the exploratory examination of the association between specific TRAEs and efficacy outcomes, secondary analyses included the assessment of efficacy outcomes, including observed response rate (ORR), progression‐free survival (PFS), and OS in patients with vs. without TRAEs of special interest—dermatologic toxicities and neuropathy. Responses were classified as complete response (CR), partial response (PR), stable disease (SD), or progression of disease (PD) and were assessed by local investigators at each site through review of clinical information and imaging reports within the EMR. Investigators were instructed to adhere as closely as possible to the Response Evaluation Criteria in Solid Tumors (RECIST v1.1) criteria, although precise tumor measurements were not obtained [25]. ORR was calculated as the sum (percentage) of CRs and PRs in the intention‐to‐treat (ITT) population. PFS was defined as the time from EV start to PD or death from any cause; alive patients without PD were censored at the date of last follow up. OS was defined as the time from EV start to death from any cause; alive patients were censored at the date of last follow up.

### 2.4 Statistical Analysis

Descriptive statistics were used to describe baseline (at EV start) clinicopathologic and TRAE characteristics and ORRs. Categorical variables, including ORR, were presented as number (percentage) and compared between groups using the Fisher exact test. Continuous variables were presented using the median value (range) and compared between groups using the two‐sided Wilcoxon rank sum test. Cox regression models were used to evaluate the effects of EV‐related dermatologic toxicities and neuropathy on time to progression and/or death, quantified as hazard ratios and associated 95% confidence intervals (CI). To control for EV treatment duration, dermatologic and neuropathy TRAEs were treated as time‐dependent covariates in the Cox regression models for PFS and OS, included at the time of occurrence following EV start. Given that the primary covariates of interest were time‐dependent and occurred at variable timepoints throughout follow‐up, survival outcomes were analyzed using time‐dependent Cox regression models, which provide a more appropriate framework than Kaplan–Meier methods for incorporating time‐dependent effects [26]. R Statistical Software version 4.1.0 was used for computation of all statistical analyses. Cox regression models were analyzed using the “survival” R package. Differences were considered significant at CIs greater than 95% (*p* < 0.05).

## 3 Results

### 3.1 Characteristics of the Study Population

This study included 485 patients with aUC who were treated with EV monotherapy between 2018 and 2023 across 16 US sites. Baseline characteristics at the start of EV for 371 patients with reported TRAEs attributed to EV and 114 patients without reported TRAEs are shown in Table 1. Of the 485 patients included in this analysis, 55 (11.3%) received EV monotherapy as part of a clinical trial; the rest as standard of care. Median time from initial UC diagnosis (localized or aUC) to EV start was 21.9 months. Median follow‐up from EV start was 9.1 months (95% CI 8.4–10.3). In the overall population, median age at EV start was 71 years (range: 35–97). Most patients were male (69.9%), White (88.2%), had primary lower tract tumors (69.7%), and pure urothelial carcinoma (68.0%). Most patients (67.0%) received two or more prior lines of therapy in the aUC setting before EV, had ECOG PS 0–1 (75.5%); prior diagnosis of diabetes mellitus and pre‐existing neuropathy were present in 18.6% and 36.7% of patients, respectively. Overall, baseline demographic and clinical characteristics at the time of EV initiation did not differ significantly between patients with and without EV TRAEs. At the time of data cutoff, 13.8% of patients remained on EV. EV was discontinued in 49.1% of patients due to PD, 21.0% due to TRAEs, 6.2% due to physician or patient decision (including transition to hospice), and 3.1% due to other medical complications unrelated to EV (Figure S4).

**TABLE 1:** Baseline characteristics of patients at start of enfortumab vedotin.

Demographic and clinical characteristics	All patients (*N* = 485)	Patients with EV TRAEs (*n* = 371)	Patients without EV TRAEs (*n* = 114)	*p*
Median age at EV start, years (range)	71 (35–97)	71 (35–97)	68.5 (37–88)	0.03
Sex, *n* (%)				
Male	339 (69.9)	252 (67.9)	87 (76.3)	0.10
Female	146 (30.1)	119 (32.1)	27 (23.7)	
Race, *n* (%)				
White	428 (88.2)	330 (88.9)	98 (86.0)	0.31
Non‐White	55 (11.3)	39 (10.5)	16 (14.0)	
Unknown	2 (0.4)	2 (0.5)	0 (0.0)	
Smoking history, *n* (%)				
Yes	302 (62.3)	233 (62.8)	69 (60.5)	0.82
No	180 (39.1)	137 (36.9)	43 (37.7)	
Unknown	3 (0.6)	1 (0.3)	2 (1.8)	
Primary tumor location, *n* (%)				
Lower tract	338 (69.7)	258 (69.5)	80 (70.2)	1.00
Upper tract	137 (28.2)	105 (28.3)	32 (28.0)	
Unknown	10 (2.1)	8 (2.2)	2 (1.7)	
Histology, *n* (%)				
Pure urothelial Variant histology	330 (68.0)149 (30.7)	247 (66.6)118 (31.8)	83 (70.2)31 (27.2)	0.35
Unknown	6 (1.2)	6 (1.6)	0 (0.0)	
Number of lines of therapy for advanced UC prior to EV, *n* (%)				
0	23 (4.7)	22 (5.9)	1 (0.9)	0.06
1	137 (28.2)	102 (27.5)	35 (30.7)	
≥ 2	325 (67.0)	247 (66.6)	78 (68.4)	
Platinum‐based therapy at any point prior to EV, *n* (%)				
Yes	300 (61.9)	222 (59.8)	78 (68.4)	0.12
No	185 (38.1)	149 (40.2)	36 (31.6)	
ICI immediately prior to EV, *n* (%)				
Yes	361 (74.4)	279 (75.2)	82 (71.9)	0.54
No	124 (25.6)	92 (0.2)	32 (28.1)	
ECOG PS, *n* (%)				
0	127 (26.2)	106 (28.6)	21 (18.4)	0.003
1	239 (49.3)	187 (50.4)	52 (45.6)	
≥ 2	98 (20.2)	63 (17.0)	35 (30.7)	
Unknown	21 (4.3)	15 (4.0)	6 (5.3)	
Prior diabetes, *n* (%)				
Yes	90 (18.6)	67 (18.1)	23 (20.2)	0.68
No	395 (81.4)	304 (81.9)	91 (79.8)	
Prior neuropathy, *n* (%)				
Yes	178 (36.7)	135 (36.4)	43 (37.7)	0.82
No	307 (63.3)	236 (63.6)	71 (62.3)	
Visceral metastases (non‐bone), *n* (%)				
Yes	289 (59.6)	206 (55.5)	83 (72.8)	0.001
No	196 (40.4)	165 (44.5)	31 (27.2)	
Liver metastases, *n* (%)				
Yes	137 (28.2)	96 (25.9)	41 (36.0)	0.04
No	348 (71.8)	275 (74.1)	73 (64.0)	
Median BMI, kg/m^2^ (range)	26.4 (15.0–51.3)	26.0 (15.0–51.3)	26.0 (16.4–50.9)	0.63
Median Hgb, g/dL (range)	11.0 (5.2–16.1)	11.3 (5.2–16.1)	9.9 (7.3–15.6)	< 0.001
Median Platelet count, K/μL (range)	253 (63–890)	253 (93–662)	258 (63–890)	0.12
Median WBC, K/μL (range)	7.56 (1.20–112.30)	7.44 (1.20–37.00)	7.94 (2.50–112.30)	0.07
Median ANC, K/μL (range)	5.31 (1.20–99.90)	5.11 (1.20–30.50)	5.64 (1.50–99.90)	0.02
Median ALC, g/dL (range)	1.00 (0.13–11.96)	1.07 (0.13–8.40)	0.90 (0.19–11.96)	0.02
Median NLR (range)	5.17 (0.00–62.67)	4.85 (0.00–46.69)	6.39 (0.39–62.68)	< 0.001
GFR, mL/min/1.73m^2^, *n* (%)				
≥ 30	443 (91.3)	339 (91.4)	104 (91.2)	1.00
< 30	34 (7.0)	26 (7.0)	8 (7.0)	
Unknown	8 (1.6)	6 (1.6)	2 (1.7)	
Median albumin, g/dL (range)	3.7 (1.2–4.8)	3.8 (1.2–4.8)	3.5 (1.9–4.7)	< 0.001

Abbreviations: ALC, absolute lymphocyte count; ANC, absolute neutrophil count; BMI, body mass index; ECOG PS, Eastern Cooperative Oncology Group performance status; EV, enfortumab vedotin; GFR, glomerular filtration rate; Hgb, hemoglobin; NLR, neutrophil: lymphocyte ratio; TRAE, treatment‐related adverse event; UC, urothelial carcinoma; WBC, white blood cell.

### 3.2 Enfortumab Vedotin TRAEs

The incidence of EV TRAEs by type and grade is summarized in Table 2. In the overall cohort, 371 (76.5%) patients were reported to have any grade TRAE attributable to EV, including 19.4% of patients with grade > 3 events. The median time to any grade TRAE onset was 4 weeks (range: 0–136). Most frequently reported any grade EV TRAEs were neuropathy (36.3%), dermatologic toxicities (26.6%), and fatigue (19.8%). Additional EV TRAEs of interest (any grade) included cytopenias (9.5%), ocular toxicities (6.0%), hyperglycemia (4.9%), and pulmonary toxicities (1.0%). Most frequent grade > 3 TRAEs were dermatologic toxicities (4.3%), neuropathy (4.1%), neutropenia (3.3%), and fatigue (2.1%). Eight patients (1.6%) experienced a fatal (grade 5) TRAE while on EV, including two cases of neutropenic fever with sepsis, two cases of sepsis unrelated to neutropenia, and one case each of infection, SJS, acute kidney injury, and encephalopathy.

**TABLE 2:** Enfortumab vedotin treatment‐related adverse events.

	Any grade	Grade 1–2	Grade ≥ 3^a^
Median time to TRAE onset, weeks (range)	4 (0–136)	4.5 (0–136)	4 (0–126)
Patients with TRAEs, *n* (%)	371 (76.5)	329 (67.8)	94 (19.4)
Neuropathy	176 (36.3)	156 (32.2)	20 (4.1)
Dermatologic toxicities	129 (26.6)	108 (22.3)	21 (4.3)
Rash	114 (23.5)	101 (20.8)	13 (2.7)
Dermatitis, blistering, toxic erythema, SJS, TEN	18 (3.7)	10 (2.1)	8 (1.6)
Fatigue	96 (19.8)	86 (17.7)	10 (2.1)
Nausea/vomiting	50 (10.3)	47 (9.7)	6 (1.2)
Cytopenias	46 (9.5)	30 (6.2)	19 (3.9)
Neutropenia (including neutropenic fever)	29 (6.0)	15 (3.1)	16 (3.3)
Anemia	16 (3.3)	12 (2.5)	4 (0.8)
Thrombocytopenia	6 (1.2)	5 (1.0)	1 (0.2)
Diarrhea	36 (7.4)	33 (6.8)	3 (0.6)
Dysgeusia/taste changes	34 (7.0)	34 (7.0)	0 (0.0)
Anorexia/weight loss	34 (7.0)	31 (6.4)	3 (0.6)
Ocular toxicities	29 (6.0)	29 (6.0)	0 (0.0)
Elevated LFTs	29 (6.0)	26 (5.4)	3 (0.6)
Hyperglycemia	24 (4.9)	16 (3.3)	8 (1.6)
Alopecia/hair loss	17 (3.5)	17 (3.5)	0 (0.0)
Pruritus	15 (3.1)	15 (3.1)	0 (0.0)
Other skin toxicities (dry skin, hyperpigmentation, skin irritation)	11 (2.3)	11 (2.3)	0 (0.0)
Electrolyte abnormalities	7 (1.4)	6 (1.2)	2 (0.4)
Renal insufficiency/AKI	10 (2.1)	4 (0.8)	6 (1.2)
Infection	10 (2.1)	4 (0.8)	6 (1.2)
Other metabolic abnormalities	5 (1.0)	4 (0.8)	2 (0.4)
Constipation	7 (1.4)	7 (1.4)	0 (0.0)
Pulmonary toxicities	5 (1.0)	4 (0.8)	1 (0.2)
Other	42 (8.7)	35 (7.2)	8 (1.6)

*Note:* This table reports the number of patients who had one or more of the specified TRAEs. Some patients may have had multiple occurrences of a single TRAE. Of 371 patients with TRAEs, 220 experienced more than one type of TRAE.

Abbreviations: AKI, acute kidney injury; LFTs, liver function tests; SJS, Stevens‐Johnson Syndrome; TEN, toxic epidermal necrolysis; TRAE, treatment‐related adverse event.

^a^ Grade 5 events occurred in 8 patients and included acute kidney injury (1), encephalopathy (1), infection (1), neutropenic fever (1), neutropenic sepsis (1), sepsis (2), and Stevens‐Johnson Syndrome (1).

### 3.3 Enfortumab Vedotin Treatment Modifications due to TRAEs

The frequency and time to treatment modification (dose reduction, interruption, or discontinuation) due to the most common EV TRAEs are shown in Table 3. Among all 485 patients, 275 patients (56.7%) experienced a TRAE leading to treatment modification. Any grade TRAEs led to drug interruption in 30.3% of patients, dose reduction in 29.9% of patients, and discontinuation of EV in 21.0% of patients. The most frequent TRAEs leading to any modification were neuropathy (24.1%), dermatologic toxicities (14.0%), and fatigue (11.5%). The most common TRAEs leading to EV dose reduction were neuropathy (12.0%), dermatologic toxicities (7.2%), and fatigue (6.4%). Similarly, the most frequent TRAEs leading to drug interruption were also neuropathy (9.3%), dermatologic toxicities (7.4%), and fatigue (4.9%). The most common TRAEs leading to permanent drug discontinuation were neuropathy (9.3%), fatigue (3.1%), and dermatologic toxicities (2.5%); only a few patients discontinued EV due to other TRAEs. Among all TRAEs, median time from EV start to TRAE onset resulting in drug discontinuation was 6 weeks (range: 0–44). This varied considerably among different TRAEs, with neuropathy resulting in the longest interval between EV start and drug discontinuation (median 12 weeks).

**TABLE 3:** Treatment modifications due to enfortumab vedotin treatment‐related adverse events.

	All TRAEs	Neuro‐pathy	Dermatologic toxicities	Fatigue	Cytopenias	Anorexia/weight loss	Ocular toxicities	Hyper‐glycemia
Patients with TRAE, *n* (%)	371 (76.5)	176 (36.3)	129 (26.6)	96 (19.8)	46 (9.5)	34 (7.0)	29 (6.0)	24 (4.9)
Dose reduction due to TRAE, *n* (%)	145 (29.9)	58 (12.0)	35 (7.2)	31 (6.4)	17 (3.5)	18 (3.7)	4 (0.8)	2 (0.4)
Drug interruption due to TRAE, *n* (%)	147 (30.3)	45 (9.3)	36 (7.4)	24 (4.9)	21 (4.3)	5 (1.0)	2 (0.4)	10 (2.1)
Drug discontinua‐tion due to TRAE, *n* (%)	102 (21.0)	45 (9.3)	12 (2.5)	15 (3.1)	5 (1.0)	2 (0.4)	1 (0.2)	1 (0.2)
Any treatment modification event due to TRAE (e.g., dose reduction, drug interruption, or drug discontinuation), *n* (%)^a^	275 (56.7)	117 (24.1)	68 (14.0)	56 (11.5)	33 (6.8)	22 (4.5)	6 (1.2)	12 (2.5)
Median time to TRAE onset leading to EV discontinua‐tion, weeks (range)	6 (0–44)	12 (1–42)	4 (2–15)	4 (1–32)	2.5 (2–44)	5.5 (2–9)	N/A	N/A

Abbreviations: EV, enfortumab vedotin; TRAE, treatment‐related adverse event.

^a^ Of 371 patients with TRAEs, 220 experienced more than one type of TRAE. Some patients may have required multiple treatment modifications, but only one treatment modification event was counted per patient.

### 3.4 Frequency of Enfortumab Vedotin TRAEs by Line of Therapy

The frequency of TRAEs characterized by line of therapy is demonstrated in Table 4. Rates of any grade TRAEs were consistent across subsequent therapy lines (74.5%–76.0% for second through fourth line), though more frequently reported in patients who received EV as first‐line therapy (95.6%), a group that contained many fewer patients (23 patients—4.7% of the cohort). A similar pattern was observed for grade > 3 TRAEs. Neuropathy of any grade was the most frequent TRAE across all treatment lines and did not appear to increase in later treatment lines. Incidence of any grade dermatologic toxicities was also consistent across different EV treatment lines, though a somewhat higher frequency of more severe (grade > 3) dermatologic toxicities was noted in patients receiving EV as fourth‐line treatment or beyond (6.5%). The rates of cytopenias associated with EV did not appear to increase in later treatment lines.

**TABLE 4:** Adverse events by line of enfortumab vedotin therapy.

	EV as Line 1	EV as Line 2	EV as Line 3	EV as ≥ Line 4
Number of patients treated by each line (out of 485 pts in study), *n* (%)	23 (4.7)	137 (28.2)	217 (44.7)	108 (22.3)

	Any grade TRAEs	Grade ≥ 3 TRAEs	Any grade TRAEs	Grade ≥ 3 TRAEs	Any grade TRAEs	Grade ≥ 3 TRAEs	Any grade TRAEs	Grade ≥ 3 TRAEs
Number of patients with TRAEs by line of EV therapy, *n* (%)	22 (95.6)	9 (39.1)	102 (74.5)	26 (19.0)	165 (76.0)	36 (16.6)	82 (75.9)	20 (18.5)
*TRAEs,* *n (%)*								
Neuropathy	11 (47.8)	1 (4.3)	51 (37.2)	7 (0.7)	73 (33.6)	6 (2.8)	38 (35.2)	6 (5.6)
Dermatologic toxicities	7 (30.4)	0 (0.0)	37 (27.0)	7 (0.7)	57 (26.3)	7 (3.2)	25 (23.1)	7 (6.5)
Rash	6 (26.1)	0 (0.0)	33 (24.1)	4 (2.9)	51 (23.5)	6 (2.8)	21 (19.4)	3 (2.8)
Dermatitis, blistering, toxic erythema, SJS, TEN	1 (4.3)	0 (0.0)	4 (2.9)	3 (2.2)	7 (3.2)	1 (0.5)	6 (5.6)	4 (3.7)
Fatigue	10 (43.5)	1 (4.3)	20 (14.6)	3 (2.2)	47 (21.7)	3 (1.4)	15 (13.9)	2 (1.9)
Nausea/vomiting	5 (21.7)	1 (4.3)	13 (9.5)	2 (1.5)	26 (11.9)	3 (1.4)	6 (5.6)	0 (0.0)
Cytopenias	3 (13.0)	2 (8.7)	12 (8.8)	4 (2.9)	19 (8.8)	7 (3.2)	10 (9.3)	4 (3.7)
Neutropenia (including neutropenic fever)	2 (8.7)	2 (8.7)	6 (4.4)	4 (2.9)	13 (6.0)	5 (2.3)	6 (5.6)	3 (2.8)
Anemia	2 (8.7)	0 (0.0)	6 (4.4)	0 (0.0)	6 (2.8)	3 (1.4)	2 (1.9)	1 (0.9)
Thrombocyto‐penia	1 (4.3)	0 (0.0)	1 (0.7)	0 (0.0)	2 (0.9)	1 (0.5)	2 (1.9)	0 (0.0)
Diarrhea	2 (8.7)	0 (0.0)	7 (5.1)	0 (0.0)	23 (10.6)	3 (1.4)	3 (2.8)	0 (0.0)
Dysgeusia/taste changes	4 (17.4)	0 (0.0)	3 (2.2)	0 (0.0)	18 (8.3)	0 (0.0)	8 (7.4)	0 (0.0)
Anorexia/weight loss	5 (21.7)	1 (4.3)	13 (9.5)	2 (1.5)	9 (4.1)	0 (0.0)	6 (5.6)	0 (0.0)
Ocular toxicities	1 (4.3)	0 (0.0)	6 (4.4)	0 (0.0)	13 (6.0)	0 (0.0)	9 (8.3)	0 (0.0)
Elevated LFTs	2 (8.7)	0 (0.0)	7 (5.1)	1 (0.7)	11 (5.1)	1 (0.5)	9 (8.3)	1 (0.9)
Hyperglycemia	0 (0.0)	0 (0.0)	9 (6.6)	3 (2.2)	8 (3.7)	3 (1.4)	5 (4.6)	2 (1.9)
Alopecia/hair loss	1 (4.3)	0 (0.0)	4 (2.9)	0 (0.0)	8 (3.7)	0 (0.0)	4 (3.7)	0 (0.0)
Pruritus	1 (4.3)	0 (0.0)	6 (4.4)	0 (0.0)	5 (2.3)	0 (0.0)	3 (2.8)	0 (0.0)
Other skin toxicities (dry skin, hyperpigmentation, skin irritation)	2 (8.7)	0 (0.0)	3 (2.2)	0 (0.0)	4 (1.8)	0 (0.0)	2 (1.9)	0 (0.0)
Electrolyte abnormalities	2 (8.7)	2 (8.7)	1 (0.7)	0 (0.0)	3 (1.4)	0 (0.0)	1 (0.9)	0 (0.0)
Renal insufficiency/AKI	0 (0.0)	0 (0.0)	3 (2.2)	1 (0.7)	5 (2.3)	4 (1.8)	2 (1.9)	1 (0.9)
Infection	1 (4.3)	1 (4.3)	4 (2.9)	2 (1.5)	3 (1.4)	2 (0.9)	2 (1.9)	1 (0.9)
Other metabolic abnormalities	0 (0.0)	0 (0.0)	1 (0.7)	1 (0.7)	2 (0.9)	1 (0.5)	1 (0.9)	0 (0.0)
Constipation	1 (4.3)	0 (0.0)	1 (0.7)	0 (0.0)	1 (0.5)	0 (0.0)	4 (3.7)	0 (0.0)
Pulmonary toxicities	0 (0.0)	0 (0.0)	3 (2.2)	0 (0.0)	1 (0.5)	0 (0.0)	1 (0.9)	1 (0.9)
Other	6 (26.0)	1 (4.3)	15 (10.9)	3 (2.2)	11 (5.1)	4 (1.8)	10 (9.3)	0 (0.0)

*Note:* Of 371 patients with TRAEs, 220 experienced more than one type of TRAE.

Abbreviations: AKI, acute kidney injury; EV, enfortumab vedotin; LFTs, liver function tests; SJS, Stevens‐Johnson Syndrome; TEN, toxic epidermal necrolysis; TRAE, treatment‐related adverse event.

### 3.5 Frequency of Enfortumab Vedotin TRAEs by Type of Prior Therapy

The frequency of TRAEs characterized by type of therapy for aUC prior to EV is demonstrated in Table 5. Most patients had received both platinum‐based chemotherapy and ICI prior to EV; the most common therapy immediately prior to EV was an ICI (74.4%). The frequency of any grade EV TRAEs was similar across the different patient groups based on prior exposure to platinum‐based chemotherapy or to ICI before treatment with EV (68.1%–77.1%). The frequencies of any grade and grade > 3 neuropathy were also similar regardless of prior therapy exposure. A similar pattern was observed with any grade dermatologic toxicities attributed to EV, regardless of prior therapy exposure. Notably, the rate of grade > 3 dermatologic toxicities was somewhat higher in patients who received platinum‐based chemotherapy immediately prior to EV (9.1%), though it was consistent in patients with prior exposure to ICI. The single patient who died from a dermatologic TRAE (grade 5 SJS) received platinum‐based chemotherapy followed by two ICI‐based regimens prior to EV.

**TABLE 5:** Adverse events by type of therapy before enfortumab vedotin.

	Platinum at any point prior to EV	Platinum immediately prior to EV	ICI at any point prior to EV	ICI immediately prior to EV
Number of patients treated by each line (out of 485 pts in study), *n* (%)	300 (61.8)	66 (13.6)	441 (90.9)	361 (74.4)

	Any grade TRAEs	Grade ≥ 3 TRAEs	Any grade TRAEs	Grade ≥ 3 TRAEs	Any grade TRAEs	Grade ≥ 3 TRAEs	Any grade TRAEs	Grade ≥ 3 TRAEs
Number of patients with TRAEs by line of EV therapy, *n* (%)	222 (74.0)	51 (17.0)	45 (68.1)	12 (18.2)	340 (77.1)	85 (19.3)	279 (77.3)	65 (18.0)
*TRAEs,* *n (%)*								
Neuropathy	102 (34.0)	9 (3.0)	19 (28.8)	3 (4.5)	166 (37.6)	19 (4.3)	139 (38.5)	14 (3.9)
Dermatologic toxicities	82 (27.3)	15 (5.0)	20 (30.3)	6 (9.1)	117 (26.5)	19 (4.3)	92 (25.5)	13 (3.6)
Rash	71 (23.7)	8 (2.7)	16 (24.2)	3 (4.5)	105 (23.8)	13 (3.0)	83 (23.0)	9 (2.5)
Dermatitis, blistering, toxic erythema, SJS, TEN	13 (4.3)	7 (2.3)	6 (9.1)	3 (4.5)	15 (3.4)	6 (1.4)	10 (2.8)	4 (1.1)
Fatigue	60 (20.0)	7 (2.3)	8 (12.1)	0 (0.0)	87 (19.7)	10 (2.3)	75 (20.8)	9 (2.5)
Nausea/vomiting	31 (10.3)	1 (0.3)	10 (15.2)	0 (0.0)	41 (9.3)	5 (1.1)	35 (9.7)	5 (1.4)
Cytopenias	26 (8.7)	11 (3.7)	6 (9.1)	3 (4.5)	41 (9.3)	16 (3.6)	32 (8.9)	11 (3.0)
Neutropenia (including neutropenic fever)	18 (6.0)	9 (3.0)	5 (7.6)	3 (4.5)	25 (5.7)	13 (3.0)	19 (5.3)	9 (2.5)
Anemia	5 (1.7)	2 (0.7)	1 (1.5)	0 (0.0)	15 (3.4)	4 (0.9)	12 (3.3)	3 (0.8)
Thrombocyto‐penia	3 (1.0)	0 (0.0)	0 (0.0)	0 (0.0)	5 (1.1)	1 (0.2)	5 (1.4)	1 (0.3)
Diarrhea	21 (7.0)	3 (1.0)	4 (6.1)	0 (0.0)	34 (7.7)	3 (0.7)	30 (8.3)	3 (0.8)
Dysgeusia/taste changes	25 (8.3)	0 (0.0)	4 (6.1)	0 (0.0)	30 (6.8)	0 (0.0)	22 (6.1)	0 (0.0)
Anorexia/weight loss	14 (4.7)	0 (0.0)	2 (3.0)	0 (0.0)	29 (6.6)	3 (0.7)	24 (6.6)	3 (0.8)
Ocular toxicities	19 (6.3)	0 (0.0)	5 (7.6)	0 (0.0)	26 (5.9)	0 (0.0)	22 (6.1)	0 (0.0)
Elevated LFTs	19 (6.3)	1 (0.3)	2 (3.0)	0 (0.0)	25 (5.7)	3 (0.7)	24 (6.6)	3 (0.8)
Hyperglycemia	14 (4.7)	3 (1.0)	2 (3.0)	0 (0.0)	23 (5.2)	8 (1.8)	18 (5.0)	7 (1.9)
Alopecia/hair loss	12 (4.0)	0 (0.0)	0 (0.0)	0 (0.0)	16 (3.6)	0 (0.0)	14 (3.9)	0 (0.0)
Pruritus	10 (3.3)	0 (0.0)	1 (1.5)	0 (0.0)	15 (3.4)	0 (0.0)	13 (3.6)	0 (0.0)
Other skin toxicities (dry skin, hyperpigmentation, skin irritation)	5 (1.7)	0 (0.0)	1 (1.5)	0 (0.0)	8 (1.8)	0 (0.0)	6 (1.7)	0 (0.0)
Electrolyte abnormalities	5 (1.7)	0 (0.0)	1 (1.5)	0 (0.0)	5 (1.1)	1 (0.2)	5 (1.4)	1 (0.3)
Renal insufficiency/AKI	7 (2.3)	5 (1.7)	1 (1.5)	1 (1.5)	10 (2.3)	6 (1.4)	9 (2.5)	5 (1.4)
Infection	4 (1.3)	3 (1.0)	1 (1.5)	1 (1.5)	10 (2.3)	6 (1.4)	8 (2.2)	4 (1.1)
Other metabolic abnormalities	4 (1.3)	1 (0.3)	1 (1.5)	0 (0.0)	5 (1.1)	2 (0.5)	2 (0.6)	2 (0.6)
Constipation	5 (1.7)	0 (0.0)	1 (1.5)	0 (0.0)	6 (1.4)	0 (0.0)	4 (1.1)	0 (0.0)
Pulmonary toxicities	3 (1.0)	1 (0.3)	1 (1.5)	0 (0.0)	4 (0.9)	1 (0.2)	4 (1.1)	1 (0.3)
Other	18 (6.0)	3 (1.0)	7 (10.6)	1 (1.5)	31 (7.0)	5 (1.1)	27 (7.5)	5 (1.4)

*Note:* Of 371 patients with TRAEs, 220 experienced more than one TRAE.

Abbreviations: AKI, acute kidney injury; EV, enfortumab vedotin; ICI, immune checkpoint inhibitor; LFTs, liver function tests; SJS, Stevens‐Johnson Syndrome; TEN, toxic epidermal necrolysis; TRAE, treatment‐related adverse event.

### 3.6 Efficacy Outcomes in Patients With EV‐Related Dermatologic Toxicities

Efficacy outcomes were compared between 129 patients who developed any grade dermatologic TRAEs and 356 who did not (Table 6). Baseline characteristics at the start of EV were similar between groups (Table S1). Median time from EV start to onset of dermatologic TRAEs was 4 weeks (range: 1–52). ORR was higher in patients who experienced EV‐related dermatologic toxicities (55.8% vs. 41.9%; *p* = 0.007). After adjusting for time on treatment with EV, ECOG PS, Hgb level, and presence of liver metastases (Table 7), dermatologic toxicities were not associated with PFS (HR 0.95; 95% CI 0.73–1.22; *p* = 0.70) or OS (HR 0.82; 95% CI 0.62–1.09; *p* = 0.20).

### 3.7 Efficacy Outcomes in Patients With EV‐Related Neuropathy

Efficacy outcomes were also compared between 176 patients who developed any grade EV‐related neuropathy and 309 patients who did not (Table 6). Patients who developed neuropathy TRAEs were less likely to have pre‐existing diabetes mellitus (12.5% vs. 87.5%; *p* < 0.0001), but more likely to have pre‐existing neuropathy (43.7% vs. 32.7%; *p* = 0.02) at the time of EV start (Table S2). Median time from EV start to onset of any grade neuropathy TRAEs was 11 weeks (range: 1–97). ORR was higher in patients who experienced EV‐related neuropathy (63.6% vs. 35.3%; *p* < 0.0001). EV‐related neuropathy was not associated with prolonged PFS (HR 0.99; 95% CI 0.77–1.26; *p* > 0.90), but was independently associated with longer OS compared to patients without EV‐related neuropathy (HR 0.66; 95% CI 0.50–0.87; *p* = 0.002) after adjusting for time on treatment with EV, ECOG PS, Hgb level, and presence of liver metastases (Table 7).

**TABLE 6:** Observed response rate in patients with vs. without any grade dermatologic or neuropathy TRAEs.

	Dermatologic TRAEs^a^			Neuropathy TRAEs^b^		
	Patients with dermatologic TRAEs (*n* = 129)	Patients without dermatologic TRAEs (*n* = 356)	*p*	Patients with neuropathy TRAEs (*n* = 176)	Patients without neuropathy TRAEs (*n* = 309)	*p*
Median time on EV, months (range)	5.9 (0.4–19.3)	4.3 (0.2–24.6)	N/A	5.9 (0.5–24.6)	3.7 (0.2–22.8)	N/A
ORR in ITT population, *n* (%)	72 (55.8)	149 (41.9)	0.007	112 (63.6)	109 (35.3)	< 0.0001
CR	8 (6.2)	19 (5.3)	0.63	15 (8.5)	12 (3.9)	0.04
PR	64 (49.6)	130 (36.5)	0.01	97 (55.1)	97 (31.4)	< 0.0001
SD	30 (23.3)	79 (22.2)	0.81	45 (25.6)	64 (20.7)	0.26
PD	15 (11.6)	86 (24.1)	0.002	19 (10.8)	82 (26.5)	< 0.0001
NE	12 (9.3)	42 (11.8)	0.51	0 (0)	54 (17.5)	< 0.0001

*Note:* For patients with multiple instances of the same TRAE (*n* = 8 for dermatologic, *n* = 5 for neuropathy), earliest time of onset was used in calculating the median time to onset.

Abbreviations: CR, complete response; EV, enfortumab vedotin; ITT, intention‐to‐treat; NE, not evaluable; ORR, observed response rate; PD, progressive disease; PR, partial response; SD, stable disease; TRAE, treatment‐related adverse event.

^a^ Median time to dermatologic TRAE onset from EV start (any grade), weeks (range): 4 (1–52).

^b^ Median time to neuropathy TRAE onset from EV start (any grade), weeks (range): 11 (1–97).

**TABLE 7:** Multivariable Cox regression models for progression‐free survival and overall survival in patients with versus without any grade dermatologic or neuropathy TRAEs.^a^

Characteristic	PFS		OS	
	HR (95% CI)	*p*	HR (95% CI)	*p*
*Dermatologic TRAEs*				
Dermatologic TRAEs (time‐dependent): yes versus no	0.95 (0.73–1.22)	0.70	0.82 (0.62–1.09)	0.20
ECOG PS				
1 versus 0	1.04 (0.80–1.34)	0.30	1.08 (0.80–1.45)	0.14
≥ 2 versus 0	1.27 (0.93–1.73)		1.40 (0.99–1.97)	
Hemoglobin: per 1 unit g/dL increase	0.87 (0.82–0.92)	< 0.001	0.84 (0.78–0.90)	< 0.001
Liver metastases: yes versus no	1.39 (1.10–1.74)	0.006	1.43 (1.11–1.84)	0.007
*Neuropathy TRAEs*				
Neuropathy TRAEs (time‐dependent): yes versus no	0.99 (0.77–1.26)	> 0.90	0.66 (0.50–0.87)	0.002
ECOG PS				
1 versus 0	1.04 (0.80–1.34)	0.30	1.05 (0.78–1.42)	0.20
≥ 2 versus 0	1.27 (0.93–1.73)		1.32 (0.94–1.87)	
Hemoglobin: per 1 unit g/dL increase	0.87 (0.82–0.92)	< 0.001	0.84 (0.79–0.90)	< 0.001
Liver metastases: yes versus no	1.38 (1.10–1.74)	0.006	1.43 (1.11–1.84)	0.007

Abbreviations: CI, confidence interval; ECOG PS, Eastern Cooperative Oncology Group performance status.; HR, hazard ratio; OS, overall survival; PFS, progression‐free survival; TRAE, treatment‐related adverse event.

^a^ Dermatologic and neuropathy TRAEs were treated as time‐dependent covariates in the Cox regression models for PFS and OS, included at the time of TRAE occurrence following EV start. Cox regression models were additionally adjusted for ECOG PS, hemoglobin level, and presence of liver metastases.

## 4 Discussion

EV has emerged as a transformative therapeutic agent in the treatment of aUC, but treatment‐related toxicities frequently limit treatment duration and can impact patients' quality of life [5, 16]. As the use of EV expands across urothelial cancer treatment settings, understanding the incidence, timing, and management of its associated toxicities is paramount. To date, these aspects of EV TRAEs have not been fully characterized in a broad population of patients outside of a clinical trial setting. In attempting to address this knowledge gap, this retrospective study of EV‐treated patients across 16 U.S. sites described the toxicity profile of EV, characterized the incidence of treatment modifications resulting from EV TRAEs, and investigated patient and treatment characteristics associated with the development of EV TRAEs. Furthermore, this analysis explored efficacy outcomes of EV in patients with two of the most frequent and dose‐limiting TRAEs, namely dermatologic toxicities and neuropathy, while accounting for EV treatment duration and known adverse prognostic factors in aUC.

In our analysis, while any grade (76.5%) and grade > 3 (19.4%) EV‐related TRAEs were common, their reported frequency was lower than in clinical trials of EV. In EV‐301, any grade and grade > 3 TRAEs occurred in 93.9% and 52.4% of patients, respectively, likely owing to more stringent and consistent TRAE monitoring and reporting in the setting of a prospective clinical trial [5, 16]. Peripheral neuropathy and dermatologic toxicities were two of the most frequent TRAEs of any grade observed in both our study and in EV‐301 [5, 16]. The overall EV toxicity profile observed in our study was comparable to previously reported data from the pivotal clinical trials of EV, although a somewhat higher percentage of patients discontinued EV due to TRAEs in this study (21%) than in EV‐301 (14%) [5, 15, 16, 27].

Peripheral neuropathy from EV is a later‐onset, dose‐related toxicity hypothesized to be related to the microtubule‐disrupting properties of the MMAE payload [28, 29, 30]. In our cohort, EV‐related neuropathy resulted in treatment discontinuation in 9.3% of patients, while this occurred in 6.0% in the EV‐301 trial [5, 16]. This may have been influenced by a higher rate of baseline neuropathy at the time of EV start in our cohort (36.7%) vs. 18.6% in the EV‐301 trial, which excluded patients with baseline grade > 2 peripheral neuropathy [5, 16]. While platinum‐based regimens are generally associated with the development of peripheral neuropathy over time [31], we did not observe an effect on EV‐associated neuropathy based on prior exposure to platinum‐based regimens in this analysis. It is likely that patients who were previously treated with platinum‐based regimens either may not have developed significant neuropathy or may not have had baseline neuropathy severe enough to preclude clinicians from offering EV. Ultimately, the higher rate of EV discontinuation outside clinical trials may reflect a patient population harboring more comorbidities as well as less familiarity or experience of treating clinicians with recommended treatment modifications to mitigate the development of EV‐related neuropathy.

The median time from EV start to neuropathy onset in our cohort was 11 weeks (range: 1–97), consistent with that reported in the EV‐301 trial [12.2 weeks (range: 0.1–56.7)] [5, 16]. We observed a previously unreported association between EV‐related neuropathy and improved efficacy outcomes, while controlling for treatment duration and known adverse prognostic factors in aUC. Preclinical studies suggest a potential association between toxicity and efficacy with ADCs related to the bystander and off‐target effects of the cytotoxic payload, largely driven by the specific linker‐cytotoxic payload composition [29, 30, 32, 33]. EV's hydrophobic cleavable linker and cell‐permeable MMAE payload may contribute to these effects, as demonstrated in both in vitro and in vivo preclinical urothelial carcinoma models [29, 33]. These findings are hypothesis‐generating and require further validation, as the complex mechanisms underlying the association between EV toxicity and efficacy remain to be elucidated. However, these reported findings may have even greater relevance for the subset of patients who develop neuropathy earlier in their treatment course with EV, since onset of neuropathy may be a potential marker of treatment response and suggest improved long‐term clinical outcomes for these patients.

Dermatologic toxicities of any grade were the second most frequent EV TRAE, occurring early in the treatment course and in 27% of our cohort vs. 47% reported in the clinical trial setting [5, 16]. The median time from EV start to EV‐related skin toxicities in our study was also slightly longer—4 weeks (range: 1–52) – than in the EV‐301 clinical trial, which had a median time to onset of 1.9 weeks (range: 0.1–55.1) [5, 16]. We hypothesize that certain low‐grade dermatologic toxicities occurring early in the treatment course are closely monitored and better captured in clinical trials, yet are likely underreported outside of the clinical trial setting, as described in similar studies examining toxicity reporting outside of clinical trial settings [34, 35].

Dermatologic toxicities are notable EV TRAEs previously described in clinical trials and real‐world analyses of EV [16, 21, 29, 36, 37]. While largely thought to represent an on‐target, off‐tumor effect due to Nectin‐4 expression on skin cells, dermatologic toxicities have been frequently observed with other MMAE payload‐containing ADCs, such as brentuximab vedotin and polatuzumab vedotin, suggesting a possible role of MMAE in their pathogenesis [29, 36, 37, 38, 39]. EV‐related skin toxicities may range from cutaneous rash to severe reactions, such as SJS/TEN [28, 36, 40]. In the long‐term follow‐up of the EV‐301 trial, any grade and grade > 3 severe cutaneous adverse reactions were reported in 20.3% and 5.1% of patients treated with EV, respectively, although these included other severe skin reactions in addition to toxic skin eruptions [16]. A post‐marketing analysis of EV identified eight cases of SJS/TEN with an estimated reporting rate of 200 out of 1,000,000 patients [41]. In our cohort, any grade severe skin reactions, including SJS/TEN occurred in 3.7% of patients, and grade > 3 severe skin toxicities occurred in 1.6% (8 patients), with one death due to SJS. Although infrequent, our observation highlights the importance of education as well as proactive and vigilant monitoring for rare but severe dermatologic TRAEs, such as SJS/TEN.

Given the expression of Nectin‐4 in human skin, EV‐related dermatologic toxicity has been hypothesized to be a potential biomarker of EV efficacy. In a single‐institution retrospective study of 51 patients with aUC treated with EV, patients with EV‐related cutaneous toxicities demonstrated a higher response rate (57.7% vs. 24.0%; *p* = 0.01), which may be influenced by EV dosing and exposure [21]. In concordance with this data, we observed a higher ORR in patients who experienced EV‐related dermatologic toxicities. Although not meeting pre‐specified criteria for statistical significance, there was also a trend toward longer OS in patients with dermatologic TRAEs that should be explored in larger and prospective cohorts. Since dermatologic toxicity occurs early in the EV treatment course and rarely necessitates treatment discontinuation if properly managed, its incidence may serve as a potential biomarker of enhanced response to EV upon further validation in large, prospective studies. In contrast, data on patient and treatment characteristics that may predict the onset of EV‐related skin toxicity are mixed. A single‐institution retrospective study of 52 EV‐treated patients with aUC, in which 48% of patients developed dermatologic toxicities found prior therapy with ICIs to be associated with increased risk for EV‐related dermatologic toxicities [42]. In our study, the incidence of EV‐related dermatologic toxicities did not differ significantly by the type of therapy given prior to EV.

Other less common EV TRAEs of special interest, such as hyperglycemia, ocular toxicities, and pulmonary toxicities were observed less frequently in our cohort than in the EV‐301 clinical trial, but nonetheless represent idiosyncratic toxicities of EV that warrant close vigilance [5, 12, 16].

This multicenter retrospective cohort study includes patients whose medical comorbidities may have led to their exclusion from investigational trials of EV, offering data more representative of a “real‐world” setting, and representing one of the largest cohorts of patients treated with EV outside of a clinical trial setting to date. Despite these strengths, there are several limitations inherent to the study design. The retrospective design of this study introduces the potential for missing or unknown data, recall bias, immortal time bias, selection bias, and residual confounding. Moreover, unlike highly controlled clinical trial settings, TRAEs in real‐world practice may be substantially underreported due to variability in clinician and patient familiarity with drug‐specific TRAEs, as well as inconsistent and nonuniform monitoring and reporting of TRAEs. Precise tumor measurements and central review of imaging were not performed, while imaging intervals among institutions and providers were not uniform, thus introducing potential variability in reporting efficacy endpoints. In comparing survival outcomes between patients with and without dermatologic toxicities and neuropathy, we attempted to control for length of EV exposure and minimize the risk of immortal time bias by performing Cox regression models using a time‐dependent covariate. However, data on other potential confounding variables were unavailable for inclusion in these models. In particular, the availability of only a single baseline weight at EV initiation and the absence of granular EV dosing data across the treatment course limit the ability to fully interpret potential exposure–related effects in this study. Finally, most patients in our cohort were White and male. Since TRAE frequency and reporting may also be influenced by differences in patient sex, race, ethnicity, and cultural factors, future studies can be strengthened by evaluating the toxicity profile of EV in larger, more diverse cohorts [43].

Recent exposure‐response and exposure‐safety analyses from landmark EV monotherapy clinical trials have advanced understanding of the relationship between EV exposure and efficacy, as well as the importance of treatment modifications in managing EV‐related toxicities without compromising efficacy [20]. However, the direct relationship between safety and efficacy remains uncharacterized. In the absence of validated biomarkers of response to EV, prospective studies are warranted to further explore these potential associations. Future investigations should also aim to identify host‐related factors that may place patients at a higher risk of developing specific TRAEs, thereby facilitating proactive mitigation strategies. Evaluation of clinical and molecular biomarkers associated with the development of EV TRAEs in the UNITE study is currently ongoing and will be reported in a subsequent analysis [44, 45]. With the FDA approval of EV in combination with pembrolizumab, the higher incidence of toxicities reported with this regimen compared to EV monotherapy, and the potential for overlapping toxicities [8, 9], further studies are needed to characterize its toxicity profile in larger real‐world cohorts and to assess the impact on treatment modifications and efficacy outcomes.

## 5 Conclusions

This large, retrospective multicenter analysis from the UNITE study characterized the toxicity profile of EV, treatment modification patterns due to EV TRAEs, characteristics associated with the development of EV TRAEs, and efficacy outcomes associated with EV‐related dermatologic toxicities and neuropathy. The toxicity profile of EV in this cohort was consistent with previously reported patterns in investigational trials of EV monotherapy. EV treatment modifications, including discontinuation, occurred most frequently due to neuropathy, dermatologic toxicities, and fatigue. EV‐related dermatologic toxicities and neuropathy were associated with a higher probability of response to EV, while only neuropathy was independently associated with longer OS after controlling for EV treatment duration and known poor prognostic factors in aUC. As the use of EV‐based regimens is expected to extend to earlier urothelial cancer treatment settings, timely identification and mitigation of EV TRAEs will be critical for optimizing clinical outcomes.

## Author Contributions

**Amanda Nizam:** conceptualization (lead), data curation (lead), formal analysis (lead), investigation (lead), methodology (lead), project administration (lead), supervision (lead), validation (lead), visualization (lead), writing – original draft (lead), writing – review and editing (equal). **Charles B. Nguyen:** conceptualization (lead), data curation (equal), formal analysis (lead), investigation (lead), methodology (lead), project administration (equal), supervision (equal), validation (lead), visualization (equal), writing – original draft (lead), writing – review and editing (equal). **Jinju Li:** formal analysis (lead), methodology (equal), software (lead), writing – review and editing (equal). **Emily C. Zabor:** formal analysis (lead), methodology (lead), software (lead), writing – review and editing (equal). **Pavlos Msaouel:** data curation (equal), formal analysis (equal), methodology (equal), writing – review and editing (equal). **Cindy Y. Jiang:** data curation (equal), writing – review and editing (equal). **Omar Alhalabi:** conceptualization (equal), data curation (equal), methodology (equal), writing – review and editing (equal). **Eugene Oh:** data curation (equal), writing – review and editing (equal). **Matthew P. Davidsohn:** data curation (equal), writing – review and editing (equal). **Ilana B. Epstein:** data curation (equal), writing – review and editing (equal). **Dimitra Rafailia Bakaloudi:** data curation (equal), writing – review and editing (equal). **Rafee Talukder:** data curation (equal), writing – review and editing (equal). **Tanya Jindal:** data curation (equal), writing – review and editing (equal). **Amy K. Taylor:** data curation (equal), writing – review and editing (equal). **Michael J. Glover:** data curation (equal), writing – review and editing (equal). **Ali Raza Khaki:** conceptualization (equal), data curation (equal), methodology (equal), writing – review and editing (equal). **Emily Lemke:** data curation (equal), writing – review and editing (equal). **Hannah Mabey:** data curation (equal), writing – review and editing (equal). **Bashar Abuqayas:** data curation (equal), writing – review and editing (equal). **Albert Jang:** data curation (equal), writing – review and editing (equal). **Jason R. Brown:** data curation (equal), writing – review and editing (equal). **Sean T. Evans:** data curation (equal), writing – review and editing (equal). **Cameron Pywell:** data curation (equal), writing – review and editing (equal). **Arnab Basu:** conceptualization (equal), data curation (equal), methodology (equal), writing – review and editing (equal). **Mehmet A. Bilen:** conceptualization (equal), data curation (equal), methodology (equal), writing – review and editing (equal). **Pedro C. Barata:** conceptualization (equal), data curation (equal), methodology (equal), writing – review and editing (equal). **Yousef Zakharia:** conceptualization (equal), data curation (equal), methodology (equal), writing – review and editing (equal). **Matthew I. Milowsky:** conceptualization (equal), data curation (equal), methodology (equal), writing – review and editing (equal). **Deepak Kilari:** conceptualization (equal), data curation (equal), methodology (equal), writing – review and editing (equal). **Christopher J. Hoimes:** conceptualization (equal), data curation (equal), methodology (equal), writing – review and editing (equal). **Sumit A. Shah:** conceptualization (equal), data curation (equal), methodology (equal), writing – review and editing (equal). **Hamid Emamekhoo:** conceptualization (equal), data curation (equal), methodology (equal), writing – review and editing (equal). **Nancy B. Davis:** conceptualization (equal), data curation (equal), methodology (equal), writing – review and editing (equal). **Shilpa Gupta:** conceptualization (equal), data curation (equal), methodology (equal), writing – review and editing (equal). **Petros Grivas:** conceptualization (equal), data curation (equal), methodology (equal), writing – review and editing (equal). **Joaquim Bellmunt:** conceptualization (equal), data curation (equal), methodology (equal), writing – review and editing (equal). **Matthew T. Campbell:** conceptualization (equal), data curation (equal), methodology (equal), writing – review and editing (equal). **Ajjai S. Alva:** conceptualization (equal), data curation (equal), methodology (equal), project administration (equal), writing – review and editing (equal). **Vadim S. Koshkin:** conceptualization (lead), data curation (equal), formal analysis (equal), methodology (equal), project administration (equal), supervision (lead), validation (equal), visualization (supporting), writing – review and editing (equal).

## Ethics Statement

This study adhered to the Declaration of Helsinki principles and received ethical approval from the institutional review boards of all participating institutions. This retrospective study was deemed minimal risk research for which a waiver for informed patient consent was granted. Deidentified data were collected following each institution's institutional review board‐approved protocol and entered in a secure central REDCap database housed at the University of Michigan in compliance with institutional IRB and legal data use agreements.

## Conflicts of Interest

Amanda Nizam reports the following disclosures—consulting or advisory role: AVEO Oncology, Astellas Pharma, Seagen, Pfizer, EMD Serono/Merck KGaA, Mashup Media, IDEOlogy Health; Honoraria: Cleveland Clinic, Aptitude Health, Targeted Oncology, IntegrityCE, MECC Global Meetings, ASCO, Doximity; Travel and accommodation expenses: ASCO, MECC Global Meetings, Mashup Media; Section editor (bladder cancer): GU Oncology Now (Mashup Media). Charles B. Nguyen reports no conflicts of interest. Jinju Li reports no conflicts of interest. Emily C. Zabor reports no conflicts of interest. Pavlos Msaouel reports the following disclosures—Consulting or advisory role: Mirati Therapeutics, Bristol Myers Squibb, Exelixis, Axiom Healthcare, KCCure; Honoraria: Exelixis, Pfizer; Steering committee member: Kidney Cancer Association; Research funding: Takeda, Bristol Myers Squibb, Mirati Therapeutics, Gateway for Cancer Research; Institutional research funding: National Cancer Institute (Grant P30 CA0166672). Cindy Y. Jiang reports no conflicts of interest. Omar Alhalabi reports the following disclosures—Consulting or advisory role: Seagen, Silverback Therapeutics, Cardinal Health; Institutional research funding: AstraZeneca, Ikena Oncology, Roche/Genentech, Arcus Biosciences. Eugene Oh reports no conflicts of interest. Matthew P. Davidsohn reports no conflicts of interest. Ilana B. Epstein reports no conflicts of interest. Dimitra Rafailia Bakaloudi reports the following disclosures—Research funding: KureIt Cancer Research Award. Rafee Talukder reports no conflicts of interest. Tanya Jindal reports no conflicts of interest. Amy K. Taylor reports no conflicts of interest. Michael J. Glover reports no conflicts of interest. Ali Raza Khaki reports the following disclosures—Consulting or advisory role (all uncompensated): Janssen, Pfizer, Astellas Pharma; Institutional research funding: Janssen, 23andMe. Emily Lemke reports the following disclosures—Consulting or advisory role: AXDEV; Honoraria: AXDEV. Hannah Mabey reports no conflicts of interest. Bashar Abuqayas reports no conflicts of interest. Albert Jang reports no conflicts of interest. Jason R. Brown reports the following disclosures—Consulting or advisory role: Pfizer, AstraZeneca, EMD Serono; Speaker's Bureau: EMD Serono; Institutional research funding: Bicycle Therapeutics, Roche, Pfizer. Sean T. Evans reports no conflicts of interest. Cameron Pywell reports no conflicts of interest. Arnab Basu reports the following disclosures—Consulting or advisory role: Sanofi Aventis, EMD Serono, Bristol Myers Squibb, Pfizer, Dendreon, Astellas Pharma, Eisai, Seagen; Speaker's Bureau: Eisai; Honoraria: Eisai, Natera, Gilead Sciences, Cardinal Health; Institutional research funding: Merck, EMD Serono, Natera, Astellas Pharma, Bristol Myers Squibb, Celgene, Roche/Genentech, AVEO Oncology. Mehmet A. Bilen reports the following disclosures—Consulting or advisory role: Exelixis, Sanofi, Nektar, EMD Serono, Eisai, Janssen, Genomic Health, Pfizer, Bristol Myers Squibb, Bayer, Exelixis, Calithera Biosciences, AstraZeneca, Seagen; Institutional research funding: Bayer, Bristol Myers Squibb, Roche/Genentech, Incyte, Nektar, AstraZeneca, Tricon Pharmaceuticals, Pfizer, Seagen, Xencor, Exelixis, Advanced Accelerator Applications, Genome and Company, Peloton Therapeutics, Merck, NiKang Therapeutics. Pedro C. Barata reports the following disclosures—Consulting or advisory role: Astellas Pharma, AstraZeneca, Bayer, Eisai, Caris Life Sciences, Exelixis, Janssen, EMD Serono, Dendreon, Pfizer, Seagen, Bristol Myers Squibb, Bayer, Guardant Health, Myovant; Honoraria: UroToday, OncLive, Targeted Oncology; Research funding: AstraZeneca, Merck, Caris Life Sciences, ESSA Pharma, Myovant, Merck, Exelixis, Merus. Yousef Zakharia reports the following disclosures—Consulting or advisory role: Bristol Myers Squibb, Amgen, Roche Diagnostics, Novartis, Janssen, Eisai, Exelixis, Castle Bioscience, Genzyme Corporation, AstraZeneca, Array, Bayer, Pfizer, Clovis, EMD Serono, Myovant; Data safety monitoring committee: Janssen Research and Development; Institutional research funding: NewLinkGenetics, Pfizer, Exelixis, Eisai. Matthew I. Milowsky reports the following disclosures—Consulting or advisory role: Loxo/Lilly, G1 Therapeutics; Honoraria: Elsevier, Medscape, Research to Practice; Stock and other ownership interests: Pfizer, Merck, Gilead Sciences; Institutional research funding: Merck, Roche/Genentech, Bristol Myers Squibb, Mirati Therapeutics, Incyte, Seagen, G1 Therapeutics, Alliance Foundation Trials, Alliance for Clinical Trials in Oncology, Clovis Oncology, Arvinas, ALX Oncology, Loxo, Hoosier Cancer Research Network, AstraZeneca/MedImmune, Novartis, Acrivon Therapeutics, Astellas Pharma, Accuray, Boehringer Ingelheim, Amgen, Constellation Pharmaceuticals, Bayer, Prostate Cancer Clinical Trials Consortium. Deepak Kilari reports the following disclosures—Consulting or advisory role: Eisai, Exelixis, Sanofi, Merck Sharp & Dohme Inc., Myovant Sciences, Astellas Pharma, Pfizer; Speaker's Bureau: Janssen, AVEO Oncology, Seagen, Exelixis; Honoraria: MJH Life Sciences, Exelixis; Travel and accommodation expenses: Exelixis, Janssen, Astellas Pharma, AVEO Oncology; Institutional research funding: Exelixis, Genentech, Sobi, Astellas Pharma, Pfizer. Christopher J. Hoimes reports the following disclosures—Consulting or advisory role: Bristol Myers Squibb, Eisai, Prometheus, Seagen, Genentech/Roche, Merck Sharp & Dohme, 2bPrecise; Speaker's Bureau: Bristol Myers Squibb, Genentech/Roche, Astellas Pharma, Seagen, Eisai; Honoraria: Seagen; Institutional research funding: Merck Sharp & Dohme, Janssen Oncology, Novartis, Alkermes, Dynavax Technologies, Nektar, NanoCarrier, Seagen, Astellas Pharma, Bristol Myers Squibb Foundation, BioNTech SE, CRISPR Therapeutics, NeoImmuneTech, Mirati Therapeutics; Uncompensated Relationships: 2bPrecise (institutional). Sumit A. Shah reports no conflicts of interest. Hamid Emamekhoo reports the following disclosures—Consulting or advisory role: Seagen, Janssen Biotech, AVEO Oncology, Cardinal Health, Exelixis. Nancy B. Davis reports the following disclosures—Employment: Merck, Sharp & Dohme Inc.; Consulting or advisory role: Janssen Biotech; Institutional research funding: AstraZeneca, Roche, Pfizer, Merck, Incyte, Mirati Therapeutics, Seagen, Astellas Pharma, Calithera Biosciences, Immunomedics, Bristol Myers Squibb, Exelixis, Gilead Sciences. Shilpa Gupta reports the following disclosures—Consulting or advisory role: Gilead Sciences, EMD Serono, Pfizer, Merck, Foundation Medicine, Seagen, Bayer, Bristol Myers Squibb/Medarex; Speaker's Bureau: Bristol Myers Squibb, Janssen Oncology, Gilead Sciences, Seagen; Stock and other ownership interests: Moderna Therapeutics, BioNTech SE, Nektar; Institutional research funding: Bristol Myers Squibb Foundation, Merck, Roche/Genentech, EMD Serono, QED Therapeutics, Seagen, Moderna Therapeutics, Exelixis, Gilead Sciences, Novartis. Petros Grivas reports the following disclosures—Consulting or advisory role: MSD, Bristol Myers Squibb, AstraZeneca, EMD Serono, Seagen, Pfizer, Janssen, Roche, Astellas Pharma, Gilead Sciences, Silverback Therapeutics, BostonGene, Fresenius Kabi, Lucence Health, PureTech, G1 Therapeutics, Aadi Biosciences, CG Oncology, Strata Oncology, ImmunityBio, Asieris Pharmaceuticals, AbbVie; Institutional research funding: Pfizer, Bristol Myers Squibb, MSD, QED Therapeutics, GlaxoSmithKline, Mirati Therapeutics, EMD Serono, G1 Therapeutics, Gilead Sciences, Acrivon Therapeutics, ALX Oncology, Genentech. Joaquim Bellmunt reports the following disclosures—Consulting or advisory role: Pierre Fabre, Astellas Pharma, Pfizer, Merck, Genentech, Novartis, AstraZeneca/MedImmune, Bristol Myers Squibb; Honoraria: UpToDate; Travel and accommodation expenses: Pfizer, MSD Oncology, Ipsen; Patents, royalties, other intellectual property: UpToDate Bladder Cancer (section editor); Stock and other ownership interests: Rainier Therapeutics; Steering Committee Member: Genentech; Data Safety Monitoring Board: Merck, Pfizer, MSD Oncology, Genentech, AstraZeneca; Institutional research funding: Millenium, Sanofi, Pfizer, EMD Serono. Matthew T. Campbell reports the following disclosures—Consulting or advisory role: Exelixis, Eisai, Seagen; Speaker's Bureau: Curio Science, Dava Oncology, MJH Life Sciences; Institutional research funding: ApricityHealth, AstraZeneca, Exelixis, Janssen, Pfizer, Seagen, United States Department of Defense. Ajjai S. Alva reports the following disclosures—Consulting or advisory role: AstraZeneca, Bristol Myers Squibb, Merck, Pfizer; Travel and accommodation expenses: Bristol Myers Squibb, Merck; Institutional research funding: Arcus Biosciences, Astellas Pharma, AstraZeneca, Bayer, Bristol Myers Squibb, Celgene, Genentech, Merck Sharp & Dohme Inc., Janssen, Mirati Therapeutics, Progenics, Prometheus Laboratories, Roche. Vadim S. Koshkin reports the following disclosures—Consulting or advisory role: Janssen, Clovis Oncology, Astellas Pharma, Seagen, Pfizer, EMD Serono, Gerson Lehrman Group, ExpertConnect, Guidepoint Global; Steering Committee Member: Seagen; Institutional research funding: Clovis Oncology, Nektar, Endocyte, Taiho Oncology, Merck, Gilead Sciences, Lilly, Seagen, Novartis, Prostate Cancer Foundation.

## Supporting information


**Data S1:** cam471284‐sup‐0001‐Supinfo.docx.

## Data Availability

Data are available upon reasonable request. All data relevant to the study are included in the article or uploaded as Supporting Information.
