# Supplementary material for: Treatment‐Related Adverse Events and Associated Outcomes in Patients With Advanced Urothelial Carcinoma Treated With Enfortumab Vedotin: Analysis of the UNITE Study
Source: Cancer Med. 2025 Oct 1;14(19):e71284. doi: 10.1002/cam4.71284 (PMC12486332; doi:10.1002/cam4.71284)
Supplement: Supplementary file 1 — Data S1: cam471284‐sup‐0001‐Supinfo.docx. [file CAM4-14-e71284-s001.docx]

**SUPPLEMENTAL FIGURES AND TABLES**

**Figure S1. CONSORT flow diagram for overall study**

Eligible patients:

- Diagnosis of advanced urothelial carcinoma
- Treated with at least one dose of enfortumab vedotin (EV) between 2018 and 2023

**(N=607)**

Excluded:

Patients treated with EV-based combination therapies

**(N=68)**

Patients treated with EV monotherapy

**(N=539)**

Final cohort of eligible patients:

- 371 patients with reported any grade EV TRAEs
- 114 patients without reported EV TRAEs

**(N=485)**

Excluded:

- 29 patients with adverse events not related to EV
- 25 patients with missing adverse event data

**(N=54)**

EV, enfortumab vedotin; TRAEs, treatment-related adverse events

**Figure S2. CONSORT flow diagram for study of outcomes in patients with vs without any grade dermatologic TRAEs**

Final cohort of eligible patients:

- 371 patients with reported any grade EV TRAEs
- 114 patients without reported EV TRAEs

**(N=485)**

Patients with any grade dermatologic EV TRAEs

**(N=129)**

Patients without dermatologic TRAEs:

- 242 patients with any grade non-dermatologic EV TRAEs
- 114 patients without EV TRAEs

**(N=356)**

- 117 evaluable for ORR
- 127 evaluable for PFS
- 126 evaluable for OS
- 314 evaluable for ORR
- 351 evaluable for PFS
- 351 evaluable for OS

EV, enfortumab vedotin; TRAEs, treatment-related adverse events; ORR, observed response rate; PFS, progression-free survival; OS, overall survival

**Figure S3. CONSORT flow diagram for study of outcomes in patients with vs without any grade neuropathy TRAEs**

Final cohort of eligible patients:

- 371 patients with reported any grade EV TRAEs
- 114 patients without reported EV TRAEs

**(N=485)**

Patients with any grade neuropathy EV TRAEs

**(N=176)**

- 176 evaluable for ORR
- 174 evaluable for PFS
- 173 evaluable for OS
- 255 evaluable for ORR
- 304 evaluable for PFS
- 304 evaluable for OS

Patients without neuropathy TRAEs:

- 195 patients with any grade non-neuropathy EV TRAEs
- 114 patients without EV TRAEs

**(N=309)**

EV, enfortumab vedotin; TRAEs, treatment-related adverse events; ORR, observed response rate; PFS, progression-free survival; OS, overall survival


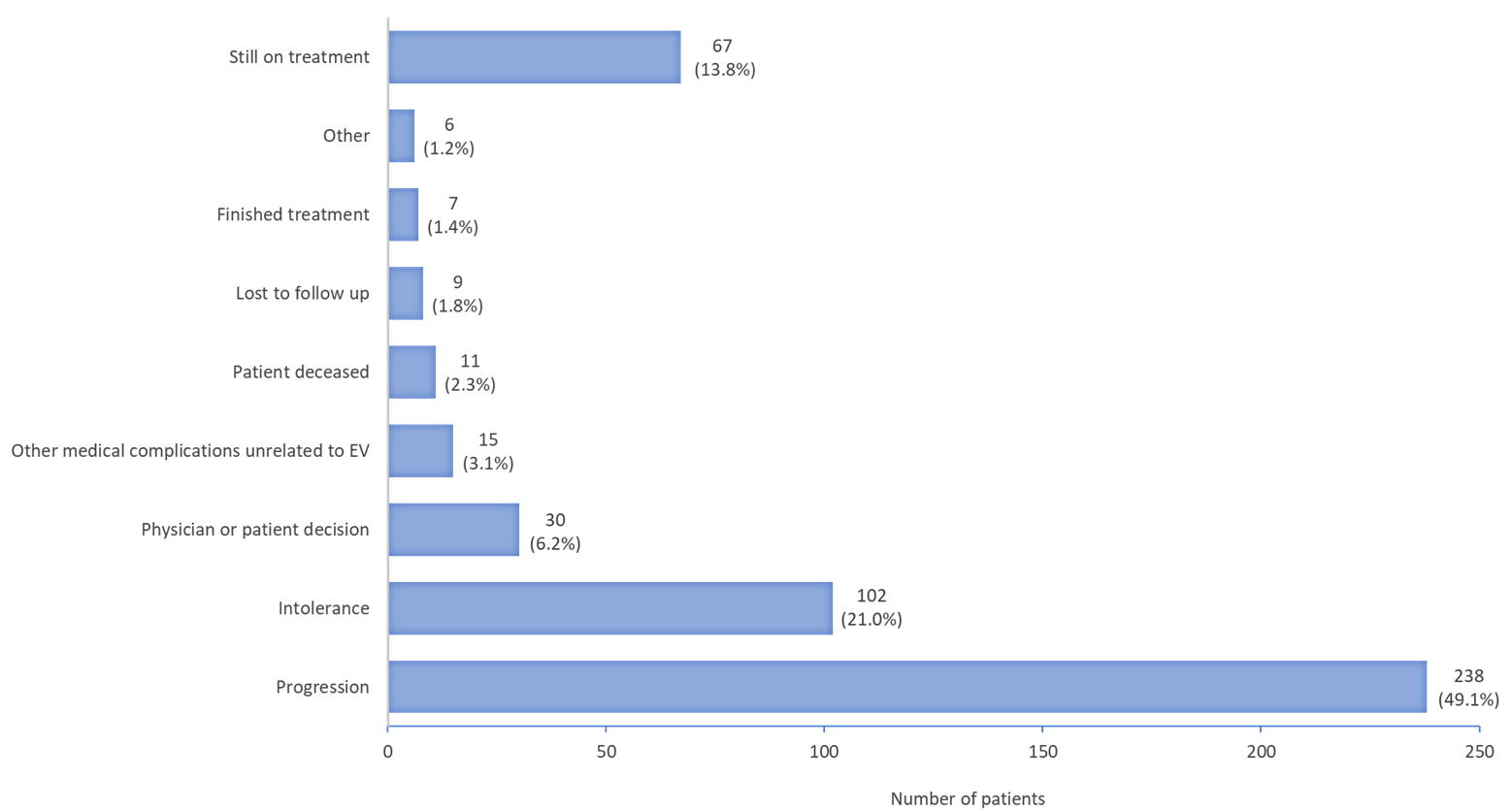
**Figure S4. Reasons for enfortumab vedotin (EV) discontinuation among all patients in the study**

| **Table S1. Baseline characteristics at the start of enfortumab vedotin in patients with vs without any grade dermatologic TRAEs** | | | | |
| --- | --- | --- | --- | --- |
| **Demographic and**  **clinical characteristics** | **All patients**  **(N=485)** | **Patients with dermatologic TRAEs**  **(n=129)** | **Patients without dermatologic TRAEs**  **(n=356)** | **p-value** |
| **Median age at EV start, years (range)** | 71  (35-97) | 72  (35-97) | 70  (37-96) | 0.07 |
| **Sex, n (%)**   - Male - Female | 339 (69.9)  146 (30.1) | 86 (66.7)  43 (33.3) | 252 (70.7)  103 (29.3) | 0.37 |
| **Race, n (%)**   - White - Non-white - Unknown | 428 (88.2)  55 (11.3)  2 (0.4) | 112 (86.8)  16 (12.4)  1 (0.8) | 316 (89.8)  39 (11.0)  1 (0.2) | 0.86 |
| **Smoking history, n (%)**   - Yes - No - Unknown | 302 (62.3)  180 (39.1)  3 (0.6) | 80 (62.0)  49 (38.0)  3 (0.0) | 222 (62.4)  131 (36.8)  3 (0.8) | 0.92 |
| **Primary tumor location, n (%)**   - Lower tract - Upper tract - Unknown | 338 (69.7)  137 (28.2)  10 (2.1) | 85 (65.9)  40 (31.0)  4 (3.1) | 253 (71.0)  97 (27.2)  6 (0.8) | 0.42 |
| **Histology, n (%)**   - Pure urothelial - Variant histology - Unknown | 330 (68.0)  149 (30.7)  6 (1.2) | 81 (62.8)  43 (33.3)  5 (3.9) | 249 (70.0)  106 (29.8)  1 (0.2) | 0.37 |
| **Number of prior lines of therapy for advanced UC prior to EV, n (%)**   - 0 - 1 - > 2 | 23 (4.7)  137 (28.2)  325 (67.0) | 7 (5.4)  37 (28.7)  85 (65.9) | 16 (4.5)  100 (28.1)  240 (67.4) | 0.89 |
| **Prior platinum-based therapy at any point, n (%)**   - Yes - No | 300 (61.9)  185 (38.1) | 82 (63.6)  47 (36.4) | 218 (61.2)  138 (38.8) | 0.67 |
| **ICI immediately prior to EV, n (%)**   - Yes - No | 361 (74.4)  124 (25.6) | 92 (71.3)  37 (28.7) | 269 (75.6)  87 (24.4) | 0.34 |
| **ECOG PS, n (%)**   - 0 - 1 - > 2 - Unknown | 127 (26.2)  239 (49.3)  98 (20.2)  21 (4.3) | 38 (29.5)  64 (49.6)  21 (16.3)  6 (4.7) | 89 (25.0)  175 (49.2)  20 (5.6)  15 (4.2) | 0.009 |
| **Prior diabetes, n (%)**   - Yes - No | 90 (18.6)  395 (81.4) | 23 (17.8)  106 (82.2) | 67 (18.8)  289 (81.2) | 0.89 |
| **Prior neuropathy, n (%)**   - Yes - No | 178 (36.7)  307 (63.3) | 51 (39.5)  78 (60.5) | 127 (35.7)  229 (64.3) | 0.45 |
| **Visceral metastases (non-bone), n (%)**   - Yes - No | 289 (59.6)  196 (40.4) | 69 (53.5)  61 (47.3) | 220 (61.8)  136 (38.2) | 0.09 |
| **Liver metastases, n (%)**   - Yes - No | 137 (28.2)  348 (71.8) | 30 (23.3)  99 (76.7) | 107 (30.1)  249 (69.9) | 0.17 |
| **Median BMI, kg/m^2^ (range)** | 26.4  (15.0-51.3) | 26.8  (16.4-47.7) | 25.98  (15.0-51.3) | 0.26 |
| **Median Hgb, g/dL (range)** | 11.0  (5.2-16.1) | 11.5  (7.7-16.1) | 10.8  (5.2-16.0) | 0.0008 |
| **Median Platelet count, K/uL (range)** | 253  (63-890) | 239  (109-556) | 258  (63-890) | 0.03 |
| **Median WBC, K/uL (range)** | 7.56  (1.20-112.30) | 6.79  (1.20-23.80) | 7.87  (2.30-112.30) | 0.0002 |
| **Median ANC, K/uL (range)** | 5.31  (1.20-99.90) | 4.40  (1.20-18.95) | 5.60  (1.33-99.90) | <0.0001 |
| **Median ALC, g/dL (range)** | 1.00  (0.13-11.96) | 1.09  (0.20-8.40) | 1.00  (0.13-11.96) | 0.29 |
| **Median NLR (range)** | 5.17  (0.00-62.67) | 3.98  (0.41-33.15) | 5.59  (0.00-62.68) | <0.0001 |
| **GFR, mL/min/1.73m^2^, n (%)**   - > 30 - < 30 - Unknown | 443 (91.3)  34 (7.0)  8 (1.6) | 122 (94.6)  6 (4.7)  1 (0.8) | 307 (86.2)  42 (11.8)  7 (2.0) | 0.02 |
| **Median albumin, g/dL (range)** | 3.7  (1.2-4.8) | 3.8  (2.3-4.6) | 3.7  (1.2-4.8) | 0.003 |
| TRAE, treatment-related adverse event; EV, enfortumab vedotin; UC, urothelial carcinoma; ECOG PS, Eastern Cooperative Oncology Group performance status; BMI, body mass index; Hgb, hemoglobin; WBC, white blood cell; ANC, absolute neutrophil count; ALC, absolute lymphocyte count; NLR, neutrophil:lymphocyte ratio; GFR, glomerular filtration rate. | | | | |

| **Table S2. Baseline characteristics at the start of enfortumab vedotin in patients with vs without any grade neuropathy TRAEs** | | | | |
| --- | --- | --- | --- | --- |
| **Demographic and**  **clinical characteristics** | **All patients**  **(N=485)** | **Patients with neuropathy TRAEs**  **(n=176)** | **Patients without neuropathy TRAEs**  **(n=309)** | **p-value** |
| **Median age at EV start, years (range)** | 71  (35-97) | 70  (44-97) | 71  (35-96) | 0.48 |
| **Sex, n (%)**   - Male - Female | 339 (69.9)  146 (30.1) | 128 (72.7)  48 (27.3) | 211 (68.3)  98 (31.7) | <0.0001 |
| **Race, n (%)**   - White - Non-white - Unknown | 428 (88.2)  55 (11.3)  2 (0.4) | 156 (88.6)  19 (10.8)  1 (0.6) | 272 (88.0)  36 (11.7)  1 (0.3) | 0.88 |
| **Smoking history, n (%)**   - Yes - No - Unknown | 302 (62.3)  180 (39.1)  3 (0.6) | 115 (65.3)  60 (34.1)  1 (0.6) | 187 (60.5)  120 (38.8)  2 (0.7) | 0.33 |
| **Primary tumor location, n (%)**   - Lower tract - Upper tract - Unknown | 338 (69.7)  137 (28.2)  10 (2.1) | 126 (71.6)  47 (26.7)  3 (1.7) | 212 (68.6)  90 (29.1)  7 (2.3) | 0.60 |
| **Histology, n (%)**   - Pure urothelial - Variant histology - Unknown | 330 (68.0)  149 (30.7)  6 (1.2) | 120 (68.2)  53 (30.1)  3 (1.7) | 210 (68.0)  96 (31.1)  3 (1.0) | 0.92 |
| **Number of prior lines of therapy for advanced UC prior to EV, n (%)**   - 0 - 1 - > 2 | 23 (4.7)  137 (28.2)  325 (67.0) | 11 (6.3)  51 (29.0)  114 (64.8) | 12 (3.9)  86 (27.8)  211 (68.3) | 0.45 |
| **Prior platinum-based therapy at any point, n (%)**   - Yes - No | 300 (61.9)  185 (38.1) | 102 (58.0)  74 (42.0) | 198 (64.1)  111 (35.9) | 0.21 |
| **ICI immediately prior to EV, n (%)**   - Yes - No | 361 (74.4)  124 (25.6) | 139 (79.0)  37 (21.0) | 222 (71.8)  87 (28.2) | 0.10 |
| **ECOG PS, n (%)**   - 0 - 1 - > 2 - Unknown | 127 (26.2)  239 (49.3)  98 (20.2)  21 (4.3) | 59 (33.5)  89 (50.6)  23 (13.1)  5 (2.8) | 68 (22.0)  150 (48.5)  75 (24.3)  16 (5.2) | 0.002 |
| **Prior diabetes, n (%)**   - Yes - No | 90 (18.6)  395 (81.4) | 22 (12.5)  154 (87.5) | 68 (22.0)  241 (78.0) | <0.0001 |
| **Prior neuropathy, n (%)**   - Yes - No | 178 (36.7)  307 (63.3) | 77 (43.7)  99 (56.3) | 101 (32.7)  208 (67.3) | 0.02 |
| **Visceral metastases (non-bone), n (%)**   - Yes - No | 289 (59.6)  196 (40.4) | 100 (56.8)  76 (43.2) | 189 (61.2)  120 (38.8) | 0.39 |
| **Liver metastases, n (%)**   - Yes - No | 137 (28.2)  348 (71.8) | 47 (26.7)  129 (73.3) | 90 (29.1)  219 (70.9) | 0.60 |
| **Median BMI, kg/m^2^ (range)** | 26.4  (15.0-51.3) | 26.1  (16.0-44.7) | 26.0  (15.0-51.3) | 0.91 |
| **Median Hgb, g/dL (range)** | 11.0  (5.2-16.1) | 11.7  (5.2-16.1) | 10.5  (6.4-15.7) | <0.0001 |
| **Median Platelet count, K/uL (range)** | 253  (63-890) | 253  (65-890) | 250  (97-539) | 0.05 |
| **Median WBC, K/uL (range)** | 7.56  (1.20-112.30) | 7.10  (2.50-34.10) | 7.84  (1.20-112.30) | 0.04 |
| **Median ANC, K/uL (range)** | 5.31  (1.20-99.90) | 4.92  (1.20-30.05) | 5.50  (1.30-99.90) | 0.05 |
| **Median ALC, g/dL (range)** | 1.00  (0.13-11.96) | 1.10  (0.20-8.40) | 0.99  (0.13-11.96) | 0.15 |
| **Median NLR (range)** | 5.17  (0.00-62.67) | 4.70  (0.41-33.15) | 5.62  (0.00-62.68) | 0.03 |
| **GFR, mL/min/1.73m^2^, n (%)**   - > 30, n (%) - < 30, n (%) - Unknown, n (%) | 443 (91.3)  34 (7.0)  8 (1.6) | 161 (91.5)  12 (6.8)  3 (1.7) | 268 (86.7)  36 (11.7)  5 (1.6) | 0.11 |
| **Median albumin, g/dL (range)** | 3.7  (1.2-4.8) | 3.9  (1.2-4.8) | 3.7  (1.9-4.7) | <0.0001 |
| TRAE, treatment-related adverse event; EV, enfortumab vedotin; UC, urothelial carcinoma; ECOG PS, Eastern Cooperative Oncology Group performance status; BMI, body mass index; Hgb, hemoglobin; WBC, white blood cell; ANC, absolute neutrophil count; ALC, absolute lymphocyte count; NLR, neutrophil:lymphocyte ratio; GFR, glomerular filtration rate. | | | | |
